# Advancing Luciferase-Based Antibody Immunoassays to Next-Generation Mix and Read Testing

**DOI:** 10.3390/bios13030303

**Published:** 2023-02-21

**Authors:** Peter D. Burbelo, Youngmi Ji, Michael J. Iadarola

**Affiliations:** 1 Adeno-Associated Virus Biology Section, National Institute of Dental and Craniofacial Research, National Institutes of Health, Bethesda, MD 202892, USA; 2 Department of Perioperative Medicine, Clinical Center, National Institutes of Health, Bethesda, MD 202892, USA

**Keywords:** antibody, autoantibody, LIPS, luciferase-based immunoassays, split luciferase

## Abstract

Antibody measurements play a central role in the diagnosis of many autoimmune and infectious diseases. One antibody detection technology, Luciferase Immunoprecipitation Systems (LIPS), utilizes genetically encoded recombinant luciferase antigen fusion proteins in an immunoglobulin capture format to generate robust antibody measurement with high diagnostic sensitivity and specificity. The LIPS technology has been highly useful in detecting antibodies for research diagnostics and the discovery of new autoantigens. The methodology of the assay requires immunoglobulin binding reagents such as protein A/G beads and washing steps to process the immune complex before antibody levels are measured by light production with a luminometer. Recently, simplified mix and read immunoassays based on split components of the nanoluciferase enzyme in a complementation format have been developed for antibody measurements without requiring immunoglobulin-capturing beads or washing steps. The mix and read immunoassays utilize two or three nanoluciferase fragments which when reconstituted via antigen-specific antibody binding generate a functional enzyme. At present, these split luciferase tests have been developed mainly for detecting SARS-CoV-2 antibodies. Here, we describe the traditional LIPS technology and compare it to the new split luciferase methodologies focusing on their technical features, strengths, limitations, and future opportunities for diagnostic research, and clinical applications.

## 1. Introduction

The detection of antibodies represents a well-established approach for the diagnosis of many infectious and autoimmune diseases. Measurements of antibodies directed against infectious agents yield not only diagnostic information for current infection but can inform about past exposure and provide detailed information about vaccine status [1]. The detection of antibodies against self-proteins, called autoantibodies, also plays a critical role in the diagnosis of many autoimmune conditions [2]. Since autoantibodies are present before the onset of clinical symptoms in many autoimmune diseases, or in some cases, directly cause the disease, detection of these autoantibodies provides unique opportunities for early clinical intervention and for monitoring therapy. Based on the clinical importance of antibody diagnostics for both infectious and autoimmune diseases, the development of simple, high-diagnostic performance immunoassays covering a large spectrum of targets is needed to meet the current and emerging healthcare demands including personalized medicine.

While many different immunoassays are available for measuring antibodies and autoantibodies including immunofluorescence, Western blot, Enzyme-linked immunosorbent assay (ELISA), protein arrays, and a variety of bead-based assays (i.e., single or multiplex assays), all these approaches have advantages and disadvantages [3,4]. By far the most widely used technique has been the ELISA, providing diagnostic results for most infectious diseases and autoimmune disorders. However, despite the wide use of ELISAs, these assays are labor intensive, require time to process, and typically measure antibodies against a single target at a time. Immunoassay technologies such as lateral flow and surface-enhanced Raman scattering (SERS)-based lateral flow tests only provide positive or negative serological status and do not yield quantitative estimates of antibody titers that are needed for vaccine monitoring and autoimmune monitoring [5]. Other less clinically developed technologies such as commercial protein arrays have the capacity to screen a large number of antigens but are not appropriate for routine diagnostics since the arrays have low sensitivity and high false positive rates. Other methods such as PhIP-Seq, an immunoprecipitation technology based on the phage-based display of large peptide libraries coupled with DNA sequencing of barcodes can be used for panoramic antibody profiling and discovery of antigens from viruses [6], bacteria [7], and humans [8]. While PhIP-Seq and other immunoassay formats have been transformative for antibody discovery, these techniques require significant expertise, equipment, and time to process the samples and are not suitable for either rapid point-of-care or routine testing.

Based on the evolving nature of multiple immunoassay formats, antibody detection technologies are on the threshold of undergoing major change, which will impact their application in clinical medicine. One first-generation non-ELISA immunoassay that employs genetically encoded luciferase fusion target proteins, Luciferase immunoprecipitation systems (LIPS), provides a highly sensitive diagnostic and discovery platform for many different antibodies [9]. More recently, several new second-generation methods based on split luciferase fusion proteins in a mix and read format have been developed that potentially can increase the utility and range of antibody testing. These new immunoassays are based on two or three split nanoluciferase recombinant fragments which, when reconstituted in the presence of specific antibodies generate measurable luciferase activity. The development of these newer split luciferase approaches suggests the potential for transformative opportunities for rapid, highly quantitative, and possibly high-capacity antibody profiling. In this review, we describe the technical details and applications of LIPS as well as the mechanics and results of the newer mix and read split luciferase assays and their potential uses for accelerating antibody measurement for clinical testing.

## 2. Luciferase Antigen Fusion Proteins for LIPS Antibody Detection

Luciferase enzymes are light-emitting reporters that are ideal for producing chimeric proteins for antibody profiling because the fusion between a potential antigen protein and the luciferase moiety retains both antigenicity and enzymatic activity, respectively. Luciferases are derived from marine plants or crustaceans and the cloning of their genes has many applications in cell and molecular biology [10]. Three luciferases, *Renilla* luciferase, *Gaussia* luciferase, and nanoluciferase, have all been utilized for measuring antibodies, and a summary of their biochemical characteristics and other attributes is shown in Table 1. The largest number of publications for antibody detection have been performed with chimeric fusion proteins with *Renilla luciferase.* This gene encodes a 36 KDa intracellular enzyme, which produces a light flash signal in the presence of coelenterazine substrate [11]. *Renilla* luciferase (Ruc) fusion proteins are well suited for tagging human, viral, and other protein antigens, in part because the fusion proteins generally exhibit a low background binding in the LIPS assay format. *Gaussia* luciferase (GLuc, from the crustacean *Gaussia princeps*), codes for a secreted protein of 185 amino acids [11]. GLuc is smaller than *Renilla* luciferase and produces a flash with the coelenterazine substrate (Table 1). When placed at the C-terminus of target proteins, GLuc produces higher luciferase activity than can be achieved with *Renilla* and often works well for detecting antibodies against secreted proteins, plasma membrane receptors, and other transmembrane proteins with extracellular regions. The most recent addition to the reporter toolbox is a luciferase derived from the small catalytic subunit of approximately 170 amino acids from the shrimp *Oplophorus gracilirostris* [12]. A transformative discovery involved changing 16 amino acids within the natural catalytic subunit of this shrimp luciferase to create a stable and highly active luciferase called nanoluciferase (Nano) [13]. Unlike the endogenous coelenterazine substrate for shrimp luciferase, nanoluciferase uses a novel furimazine reagent (Table 1). The light produced by Nano with furimazine is a long-lasting glow reaction, which can even be used to measure antibodies with a standard tube and plate luminometer, as well as a portable hand-held luminometer [14].

**Figure 1:**
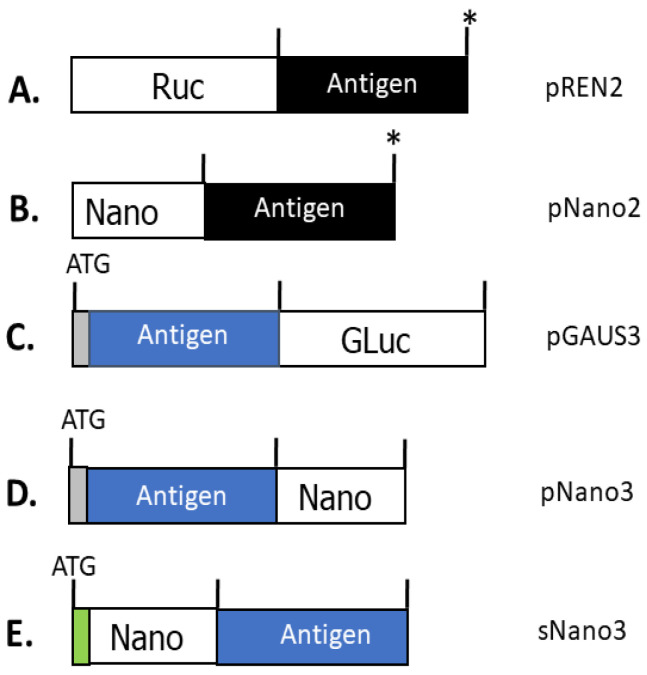
Examples of luciferase antigen fusion protein designs for LIPS. Different luciferase-antigen configurations as fusion proteins for LIPS analysis (**A**–**E**). Cytoplasmic antigens are denoted by the black rectangle along with stop codon (asterisk). Blue boxes are extracellular proteins with endogenous (gray) or heterologous (green) signal sequence.

LIPS employs recombinant luciferase-antigen fusion proteins in an immunoprecipitation format. The experimental details of the LIPS technology have been previously described [9]. Several features of LIPS, including rapid production of recombinant luciferase-antigen extract that can be used for testing without purification, high signal-to-noise ratio, and wide dynamic range of antibody detection make it ideal for antibody profiling and discovery. The fusion proteins are stable and obviously non-radioactive. Full-length proteins, protein fragments, and peptides have all been used successfully as targets in LIPS. The exact molecular design of the fusion plasmid constructs can be optimized for protein folding and expression in mammalian cells. Intracellular protein antigens generally perform well as C-terminal fusions with Ruc (Figure 1A), and Nano (Figure 1B). In some cases, such as short cytokines, the proteins can be expressed as a C-terminal fusion without the need for a signal sequence (Figure 1A,B). Larger secreted proteins and integral membrane proteins, particularly if they contain a hydrophobic signal sequence, should be configured as N-terminal fusions with GLuc (Figure 1C) or Nano (Figure 1D). With some processed secreted proteins, a vector encoding a heterologous signal sequence, such as from IL6 or PTH is placed before Nano and the antigen target is cloned at the C-terminus (Figure 1E). This vector strategy has produced properly folded secreted proteins including those for insulin [15] and the spike protein of SARS-CoV-2 [16]. Lastly, there are no strict rules, and the recommended design is meant merely as a starting point for the construction of these antigen fusions.

Following the construction of mammalian expression plasmids for luciferase-antigens, they are transfected into mammalian cells for the production of light-emitting protein extracts. Typically, mammalian cell lines such as HEK-293 and COS cells are used to efficiently make recombinant proteins, which can be monitored directly by the luciferase activity expressed in light units (LU). Producing high levels of luciferase in mammalian cells can be further enhanced by the generation of nanoluciferase-antigen fusions, which generally produce 10–100 times higher light emission than *Renilla* or *Gaussia* luciferases. Typically, two days after transfection, cells are harvested to produce crude extracts by cell scrapping in lysis buffer and centrifugation, without the need for sonication or time-consuming protein purification. The cell layer is simply scrapped in an extraction buffer containing 0.1% Triton X-100, which lyses the cells. The crude extracts are clarified by centrifugation and supernatants are assessed for input to the LIPS assay by measurement in a luminometer. Importantly, the crude extracts containing the luciferase-tagged antigens can be harvested with the addition of glycerol and stored as frozen aliquots that can be thawed for later use. This enhances the utility and standardization of the assay.

**Figure 2:**
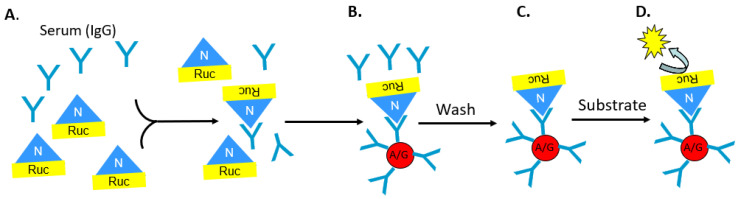
Schematic of the LIPS assay for detecting antibodies. LIPS is based on the fluid-phase capture of immunoglobulins. As shown for detecting antibodies against the SARS-CoV-2 nucleocapsid (N) protein. In the first step, (**A**) aliquots of the Ruc-SARS-CoV-2 N protein extract are incubated with serum samples and buffer. (**B**) The antibody complexes are then captured by protein A/G beads in a filter plate and (**C**) the unbound luciferase-tagged antigen is washed away. (**D**) The amount of captured antigen present is determined by adding luciferase substrate and emitted light is measured with a luminometer, which is specifically proportional to the amount of bound SARS-CoV-2 N antibody.

For LIPS antibody testing, the addition of a defined amount of the luciferase-antigen fusion protein, based on the LU of the extract, typically 10 million LU, is used per sample. To set up the assay, serum, buffer, and cell extract are combined and incubated for one hour. These assays require only 1.0 microliter of serum/plasma and between 5–10 microliters of saliva. If specific antibodies are present, they bind to the target antigen-luciferase fusion protein (Figure 2). The reaction mixture is then transferred to a filter plate containing antibody-capturing reagents such as protein A/G beads, anti-IgA beads, or other secondary immunoglobulin-immobilized beads and incubated for an additional hour. While the capture beads such as protein A/G beads can bind both free immunoglobulins and antibodies bound to the luciferase-tagged antigen, the free unbound luciferase-tagged antigen is removed from the microtiter filter plate by multiple washing steps, with the beads being retained on the plate throughout. Next, the relative amount of antibody bound in the immune complex is determined by placing the 96-well plate into the luminometer and measuring light production upon adding the appropriate coelenterazine or furimazine substrate (Figure 2). The time required to perform LIPS testing is under 2.5 h and is typically faster than ELISA and Western blotting. It should be noted that other LIPS formats can be performed to collect highly quantitative antibody data including single tube assays, rapid tests, arrays, and even a microfluidic device.

Another approach using luciferase-tagged antigens involves microtiter plates coated with Protein A to capture the antigen-antibody complexes. This plate method does not require the more expensive microtiter filter plate and has shown promise as an inexpensive way to measure antibodies for different pathogens including against HIV [17] and Zika virus [18].

## 3. LIPS Autoantibody Profiling of Autoimmune and Infectious Diseases

The detection of autoantibodies is an important clinical component for the diagnosis and monitoring of many different autoimmune diseases [2]. LIPS is ideally suited for studying autoantibodies in autoimmunity due to the relative ease in developing the required immunoassays. One advantage of LIPS over ELISA is the highly robust nature of the seropositive signals generated against various autoantigens seen in these diseases. Specifically, the LIPS-based luminometer readings often span a wide dynamic range resulting allowing easy discrimination between seropositive and seronegative signals, thereby representing ideal immunoassay tests for studying these autoantibodies in both cross-sectional and longitudinal studies. Importantly, LIPS has been used to study a number of well-known autoimmune diseases resulting in patient subclassifications and delineating temporal relationships between clinical manifestations and the appearance of detectable autoantibodies including autoimmune polyendocrinopathy-candidiasis-ectodermal dystrophy (APECED) [19], autoimmune encephalitis [20], systemic lupus erythematosus [21,22] Sjogren’s syndrome [23,24,25], biliary cirrhosis [26,27], systemic sclerosis/scleroderma [28], membranous nephropathy [29,30], and atrophic body gastric [31,32,33]. In type I diabetes (T1D), LIPS assays detect robust autoantibodies against a variety of known autoantigens including IA2, IA2-beta, and GAD65 [9], as well as establishing new immunoassays for such targets such as tetraspanin-7 [34,35,36,37] and PPIL2 and MLH1 [38]. One relatively new successful advance has been the detection of anti-insulin autoantibodies in T1D with a non-radioactive LIPS format by two groups [15,39]. Based on the autoantibody heterogeneity seen in T1D, LIPS assays provide high sensitivity and specificity non-radioactive immunoassays in a standard format for distinguishing T1D patients from controls. Based on various publications describing numerous T1D antigens in the LIPS format, studies in the future may use LIPS to profile simultaneously many of these autoantigens to further classify different T1D individuals into subsets for potentially understanding pathogenesis and progression.

Besides cross-sectional studies in autoimmunity, LIPS has been used to investigate autoantibody responses over time to understand disease progression before disease diagnosis and to monitor treatment. In one study of retrospective biobanked serum samples, LIPS was used to detect autoantibodies against Ro52, Ro60, CENP-A, and other autoantigens in subjects with systemic sclerosis years before clinical diagnosis highlighting detectable autoantibodies in some cases ~25 years before diagnosis [28]. In another study of the major pathogenic autoantibody target in membranous nephropathy, LIPS evaluation of autoantibodies against PLA2R in 50% of the cases right before clinical diagnosis, and in the other cases the antibody was present years before disease diagnosis, consistent with the flares and known spontaneous remission of the disease [30]. The antibody profiles generated by LIPS can be also used for monitoring response to treatment including in membranous nephropathy [29] highlighting the future interrogation of autoantibody profiling in the clinical management of autoimmune diseases.

LIPS also has been instrumental in the serology-enabled discovery of novel autoantibodies against proteins with extracellular domains or secreted proteins that directly cause disease including cytokines, secreted molecules, and plasma membrane receptors (Table 2). In 2010, LIPS was used to screen for the presence of autoantibodies against a panel of 15 different cytokines associated with opportunistic infection in thymoma cancer patients. This study discovered several anti-cytokine autoantibodies including against interleukin-12 (IL-12), interleukin-17 (IL-17), and interferon-α (IFN-α) in a subset of patients with opportunistic infections (OI), in which follow-up analysis showed that many of these antibodies were neutralizing thereby implicated them in the underlying opportunistic infections [40]. At the time, simultaneous measurements of autoantibodies against multiple cytokines had not been previously explored. In another study, LIPS screening of 45 different cytokines identified pathogenic autoantibodies against another major cytokine, interferon-γ, in patients with disseminated non-tuberculosis mycobacterial infection (dNTM) [41]. Here, high levels of interferon-γ autoantibodies block IFN-γ receptor signaling of this cytokine thereby causing a severe immunodeficiency leading to dNTM as well as other opportunistic infections. More recently LIPS has been used to study a variety of anti-cytokine autoantibodies including in autoimmune polyendocrinopathy-candidiasis-ectodermal dystrophy (APECED) and/or thymoma patients [42,43,44].

Other examples of LIPS-based autoimmune discovery include the discovery of the first case of autoimmune tumoral calcinosis by autoantibodies against the FGF-23 hormone that controls blood phosphate levels [45]. In addition, LIPS was used to discover the first two cases of autoimmune parathyroid hormone resistance triggered by autoantibodies against the PTH1 receptor associated with very low blood calcium [46]. These studies highlight the utility of the LIPS approach for discovering new targets of autoantibodies that directedly cause autoimmune-mediated pathogenic conditions. The major advantages of LIPS for this discovery research are the robust sensitivity and the processivity of making the antigenic targets and corresponding autoantibody measurements.

Robust antibody detection with the LIPS assay has also contributed significantly to the study of diverse infectious agents. As described, LIPS assays can detect antibodies associated with over thirty human and animal infectious diseases [9]. Since the 2015 review article, additional LIPS tests continue to be developed to detect diagnostically useful antibodies against other human and animal pathogens including Ebola virus [47], toxoplasmosis [48,49], norovirus [50], African swine fever virus [51,52], elephant endotheliotropic herpesvirus (EEHV) [53], tick-borne encephalitis virus (TBEV) [54], porcine reproductive and respiratory syndrome virus (PRRSV) [55] and Severe Fever with Thrombocytopenia syndrome virus (SFTSV) [56,57]. A LIPS test for Severe Fever with SFTSV found additional seropositive cases of this new viral disease that were not diagnosed during hospital examination [56]. In some of these studies, the detection of robust antibody responses against multiple proteins expressed by the infectious agents was used for detailed characterization of the spectrum of humoral responses.

Besides established infectious agents, there remains an increasing need to discover and characterize new human and animal pathogens. LIPS has provided an important tool for the discovery and exploration of several new viral agents. Importantly, LIPS has been used to investigate and characterize several Coronaviruses that cause severe pathology. Prior to the COVID-19 pandemic, Zhou et al. (2018) used several molecular approaches along with LIPS and identified the viral cause of a novel fatal porcine diarrhea outbreak in China [58]. In this study, the active agent identified infecting pigs was a novel coronavirus, SADS-CoV, and its genome sequence was analyzed. Based on the viral sequence of the capsid protein, a luciferase capsid fusion protein was developed and employed in LIPS testing, which established serological evidence for in vivo porcine infection. As noted by the authors, instead of classic virology to characterize the SADS-CoV, next-generation sequencing in combination with LIPS serology was employed to rapidly characterize the infectious agent as a new pathogenic coronavirus [58].

In late November of 2019, less than 2 years after the porcine SADS-CoV publication, the COVID-19 pandemic emerged. With previous virological and serological studies as a guide, at least six independent groups used LIPS to study humoral responses against SARS-CoV-2 [16,59,60,61,62]. One group utilized SARS-CoV-2 nucleocapsid and spike as *Renilla* and *Gaussia* luciferase fusions, respectively, to evaluate antibody emergence after initial COVID-19 infection in different patient groups including immunocompromised individuals [59]. In an international study, these LIPS tests were used to confirm the presence of SARS-CoV-2 antibodies to document infection status and identified autoantibodies against IFN-α and IFN-ω in a subgroup of mainly males as contributing to severe COVID-19 [63]. Lampasona’s group established dual LIPS assays for both nucleocapsid and spike proteins to study how antibodies to SARS-CoV-2 evolved over time and correlated with survival [16,64]. In another of their studies in a pediatric population, employing these dual assays identified significantly higher SARS-CoV-2 seroprevalence than reported in asymptomatic children highlighting the improved sensitivities of these assays [65]. A third group used LIPS to screen antibody responses against additional antigenic proteins from the proteome of the SARS-CoV-2 virus and identified immunoreactivity against several minor antigens including ORF8 and ORF3B as markers of early and late infection [62]. In a subsequent study, these two minor antigens were useful for identifying a distinct serological signature in children [66]. Overall, these multiple LIPS studies add to the understanding of SARS-CoV-2 infection and provide a deeper insight into the humoral responses to SARS-CoV-2 at a molecular and diagnostic level.

Another application of LIPS for SARS-CoV-2 research involves the investigation of antibody responses against spike protein vaccines. Several LIPS studies have measured spike antibody levels in patients receiving SARS-CoV-2 vaccines in vulnerable patient populations including inborn errors of immunity [67], and cancer patients receiving immunosuppressive agents such as rituximab [68,69]. Remarkably in a study of a patient with B-cell lymphoma with profound B-cell depletion after chemoimmunotherapy, vaccine-induced anti-SARS-CoV-2 spike antibodies were induced only after the fifth and sixth doses of the vaccine [69]. These LIPS studies provide insights into the efficacy of vaccine responses of unique patient groups and provide information for the management of such patients.

## 4. Split Nanoluciferase-Based Immunoassays for Antibody Detection

While LIPS in its standard configuration is a powerful tool for the discovery and diagnosis of infectious and autoimmune diseases, it has not yet been adapted to the rapid point-of-care use in the field or clinic. As an alternative strategy for assay simplification, several groups have recently exploited the unique biomarker potential of luciferases based on specific antibody-mediated reconstitution of luciferase enzyme fragments into an intact catalytically active enzyme. This complementation strategy is based on existing studies showing that all three luciferases, Ruc, GLuc, and Nano, can be split into protein fragments, and when brought into proximity, can reconstitute enzymatic activity. Initially, the applications of these “split systems” were in areas of cell biology [70]. The luciferase reporter of particular importance for antibody detection studies is Nano, which can be dissected into two and even three protein fragments, and when reconstituted, generate high enzymatic luminescent activity [71,72,73]. In the case of the two-component Nano system, there is a small fragment (SmBiT) and a large fragment (LgBiT) that when brought into proximity reconstitute enzymatic activity. There is also another version that involves the LgBiT in combination with a self-assembling short peptide called HiBiT, which together reconstitute enzymatic reporter activity. Lastly, a three-component system of Nano fragments has been developed [72]. This three-component Nano system can generate enzymatic activity when a large (LgFgt/D11S) fragment associates with two 11 amino acids peptides designated β9 polypeptide and β10 polypeptide [72,73]. The use of split luciferase fragments as antibody detectors utilizes the functional and structural characteristics of IgG molecules including the bivalent Fab arms involved in antibody binding to two target epitopes and the C-terminal Fc binding region which allows IgG molecules to bind protein A and/or G proteins. As described in the following section, several split luciferase immunoassays were developed mainly to meet the need for the point of detection of IgG antibodies against SARS-CoV-2 based on different antibody interactions that mediate enzymatic reconstitution of Nano fragments.

**Figure 3:**
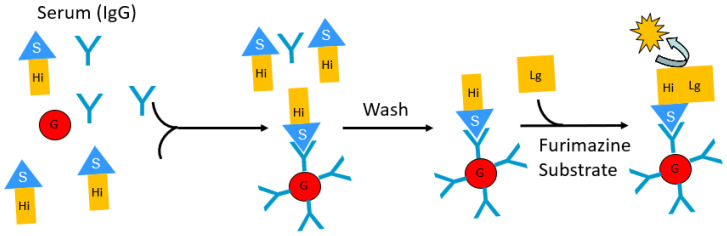
A LIPS-like split luciferase immunoassay for antibody measurements. This immunoassay format utilizes split luciferase fragments in a LIPS-like assay. As shown above, for detecting antibodies to the receptor binding domain (RBD) of SARS-CoV-2 spike protein (S), a fusion protein encoding the cDNA for RBD of the spike protein is genetically fused with the HiBiT split luciferase Nano fragment. This recombinant spike RBD-HiBiT fusion protein is incubated with patient serum samples in the presence of protein G IgG-binding beads. After washing, the non-bead-bound spike RBD-HiBiT protein is removed. Next, the LgBiT along with furimazine substrate is added. If the HiBit-SARS-CoV2 spike protein is present, the self-assembling LgBiT protein fragment binds and reconstitutes a functional Nano enzyme producing light that is measured by a luminometer. The amount of specific RBD spike antibody is proportional to the amount of bound antigen present.

As shown in Figure 3, one split Nano system, based on a modified LIPS strategy, although not technically configured in a simple mix and read format, involves a recombinant fusion protein of the SARS-CoV-2 receptor binding domain (RBD) of spike protein fused to the HiBiT fragment [74]. Both the recombinant spike RBD-HiBiT and LgBiT proteins were obtained as crude lysates from transfected mammalian cells. The assay methodology involves incubating the spike RBD-HiBiT protein with 5μL patient serum/plasma and protein G beads in a 384-well format for 30 min. If serum antibodies are present against the RBD spike-HiBiT fusion protein, the antibodies bind the spike RBD-HiBIT fusion protein, and this antibody-antigen immune complex is immunoprecipitated by protein G beads (Figure 3). After washing to remove free RBD spike-HiBiT fusion protein, the bead-bound complexes are further incubated passively with the unfused, complementary LgBiT fragment, which reconstitutes the luciferase enzyme if the HiBiT component is present. This step is carried out in the presence of furimazine Nano substrate, which generates a light signal that is proportional to the amount of antibody bound. Testing of this hybrid method showed that serum samples from uninfected individuals had low luminescence signals with no false positives, but the SARS-CoV-2-infected patients showed much higher luminescence signals. Although the authors claim the immunoassay had high sensitivity and specificity, there was limited comparison with other immunoassays. Lastly, since this split luciferase immunoassay, like LIPS, requires antibody-capturing protein G beads and washing steps, this assay is still labor intensive and, in fact, is even more complicated than the standard LIPS assay.

**Figure 4:**
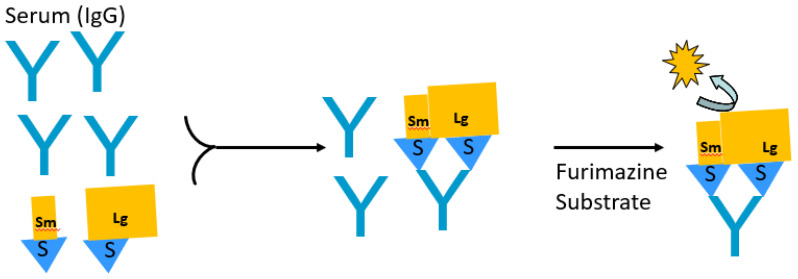
Split Nano Fab binding immunoassay for detecting antibodies. In this assay, purified antigen fusion proteins of SmBiT and LgBiT Nano fragments are incubated with serum samples. Shown is an immunoassay employing the SARS-CoV-2 spike (S) protein with the SmBit and LgBiT. If a spike antibody is present, in some cases, an antibody with one Fab arm binds to the LgBit sensor and the other Fab arm binds to the SmBit sensor. This binding of the two Fabs of a spike antibody to both the SmBit and LgBit Nano fragments reconstitutes Nano and produces a luciferase signal output, in which the amount of specific antibody present is proportional to the light produced. Antibody molecules that have bound only two molecules of SmBiT or only two molecules of LgBiT do not complement and do not produce detectable luciferase activity (not shown).

By comparison, a truly simple mix and read assay format is based on a strategy exploiting the two Fab arms of an antibody for antigen binding activity. In this assay, the two separate LgBiT and SmBiT components are genetically fused to the same target antigen and must be first purified as recombinant proteins (Figure 4). If an antibody is present, Fab binding to the spike RBD-LgBiT and spike RBD-SmBiT proteins bring the two Nano fragments into proximity and reconstitute Nano luciferase activity. Stoichiometrically this occurs only when the LgBiT and SmBiT are bound to the same IgG molecule. This assay does not require immunoglobulin-binding beads or washing steps. In one report by Elledge et al., the LgBiT and SmBiT were developed to measure antibodies against both the spike and nucleocapsid proteins of SARS-CoV-2 [14]. Antibodies against the spike protein of SARS-CoV-2 focused on the spike receptor binding domain (RBD) and were fused genetically to both the SmBiT and LgBiT protein fragments. As shown in Figure 4, the SARS-CoV-2 RBD immunoassay is initiated in solution by incubating human sera with the two RBD spike nano fragments. If an antibody is present, the two Fab arms can bring into proximity the SmBiT and LgBit, thereby reconstituting Nano luciferase activity.

The split Nano Fab-binding immunoassay could detect antibodies over a range of serum dilutions with high sensitivity and specificity in about 1 h, in which the amount of antibody present is proportional to the light produced [14]. Of note for assay set-up, the spike RBD-SmBiT and spike RBD-LgBiT fusion proteins are first incubated for 30 min before adding furimazine reagent to measure luciferase activity. A detailed protocol for detecting SARS-CoV-2 spike and nucleocapsid antibodies using this split system is provided [75]. As described, one important element of consideration of Fab-binding immunoassay is that the antigen target cannot contain a protein dimerization domain because this would allow the SmBiT and LgBiT protein fragments to reassembly Nano activity in the absence of specific antibody binding. This situation was a factor when developing a test for detecting antibodies against the nucleocapsid of SARS-CoV-2 [14]. For the nucleocapsid of SARS-CoV-2, only the N-terminal region (amino acids 44–257) was employed in this split luciferase assay format, because the nucleocapsid contains a C-terminal dimerization domain. An assay using this SARS-CoV-2 nucleocapsid region fused to the Nano LgBiT and SmBiT showed high sensitivity and specificity [14].

In a follow-up report, the split luciferase Fab-binding immunoassays for spike and nucleocapsid antibody detection were compared to a variety of immunoassays including standard LIPS, ELISA, and Luminex formats and produced extremely promising diagnostic performance equivalent to the other assays [76]. Moreover, a second independent group developed a similar split Nano strategy based on Fab binding, called Rappid, to examine SARS-CoV-2 antibodies by using SARS-CoV-2 RBD of the LgBiT and SmBiT [77]. Taken together, these studies point to the simplified and robust nature of this Nano split approach for antibody-based diagnostics. Other features of the assay were the relative stability after lyophilization and the finding that the assay could also be performed with a hand-held portable luminometer [14].

**Figure 5:**
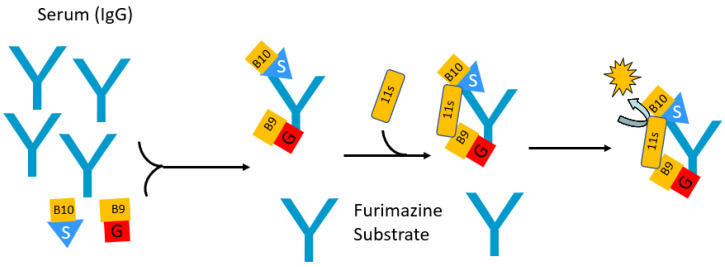
Three-component split luciferase assay (SATiN) for detecting antibodies. Three split nanoluciferase fragments are utilized. The small nanoluciferase β10 fragment is generated as a fusion protein with the target antigen. A second β9 nanoluciferase peptide is made as a fusion with the Fc immunoglobulin capturing protein G. When these two components are added with serum if the antibodies are present against the target, the Fab portion of the IgG will bind the β10-spike antigen fusion (yellow and blue) and subsequently the β9-protein-G fusion (yellow and red) will be captured by the Fc portion of the same immunoglobulin molecule. To complete the reaction a third large nanoluciferase fragment (D11S) is added. This completes the proximity bridge and upon addition of furimazine substrate produces light. Light production is proportional to amount of antibody present.

Another strategy shown in Figure 5 for measuring SARS-CoV-2 spike antibodies involves three Nano fragments for complementation that include the β9 (11 amino acids), β10 (11 amino acids), and D11S (LgFgt) protein fragments in a technique called SATiN [78]. In this assay, the β10 peptide is genetically fused to the spike RBD antigen and the β9 peptide is genetically fused to protein G. The β9 and β10 proteins fusions and a third D11S recombinant protein, all contain His-tags for purification from bacteria using affinity chromatography prior to use in the final assay. For detecting SARS-CoV-2 spike antibodies by SATiN, the chimeric fusion of β9-protein G is added with a SARS-CoV-2 spike-β10 fragment fusion protein (Figure 5). If a SARS-COV-2 specific antibody is present, it binds the β10-spike RBD probe whereby the same IgG molecule is simultaneously captured via the Fc interaction with the β9-protein G fusion protein. If β9 and β10 protein fragments are in proximity, the addition of the D11S protein fragment, allows the passive docking of the D11S “bridge” protein fragment thereby reconstituting luciferase activity. The incubation times are relatively short requiring 30 min for the first two components followed by the addition of the D11S fragment and the furimazine substrate for generating the light signal. As noted by the authors, due to non-specific antibody interference, a range of serum dilutions 1:300, 1:900, and 1:2700 are tested and averaged to approximate the correct amount of antibody present. Comparative studies showed SATiN had similar sensitivity for detecting anti-spike antibodies with an ELISA and several neutralization assays. Further development is needed for SATiN to streamline the procedure due to more complex titrations needed for the three components as well as the required testing of three different serum dilutions.

Besides detecting SARS-CoV-2 antibodies, a similar tripartite split nanoluciferase strategy has been employed to measure tumor necrosis factor (TNF) therapeutic antibodies [79]. TNF antibodies are used to treat a variety of inflammatory and autoimmune diseases. For measuring TNF antibodies in the three-component Nano system, the β9-protein A and β10-TNF genetic fusion constructs along with the LgFgt proteins were expressed in E. coli to produce the three recombinant proteins. To initiate the assay, serum samples potentially containing the anti-TNF antibody are incubated with the β9-protein A and β10-TNF antigen fusion proteins. If an antibody is present, the antibody binds to the β10-TNF protein fragment and the same antibody is captured by the β9-protein A fusion. The bound complex passively binds the LgFgt fragment to complete the full reconstituted enzyme and in the presence of furimazine substrate generates a robust, measurable light signal. The entire protocol from start to finish can be completed in under two hours with a total hands-on time of less than 5 min. Overall, this study along with SARS-CoV-2 split assays highlights the possibility of using these approaches for measuring antibody and autoantibody targets in clinical samples.

## 5. Discussion

LIPS was the first technology to exploit luciferase fusion proteins for measuring antibodies for autoimmune and infectious diseases [9]. LIPS has multiple advantages that confer specificity and rapid assay development time when approaching known or new antigens. These features include a modular defined molecular design for developing antigen fusions, simplicity in the preparation of antigen extracts, and the relatively small amount of time required for assay optimization. LIPS has been used to characterize autoantibodies in multiple autoimmune diseases and for the discovery of novel autoantibodies in several autoimmune conditions. LIPS has even been used to rule out the presence of some autoantibodies in certain diseases including against KIR4.1 in multiple sclerosis [80], IL-2 in T1D [81], and ezrin in pancreatic ductal cancer [82]. Moreover, LIPS assays have been used to detect antibodies to numerous infectious agents including the near full-proteomes of HIV, HCV, and HTLV-I [9]. The relatively modular, streamlined protocol has also made it a popular approach for detecting antibodies against the nucleocapsid, spike, and other SARS-CoV-2 proteins.

Despite the powerful capabilities of LIPS, rapid point-of-care tests developed using the technology are relatively sparse. This is mainly due to the required washing steps of the bead-bound immune complexes. The alternative strategies discussed here have been developed based on the reconstitution of split nanoluciferase protein fragments in complementation assays. The feasibility of these split Nano immunoassays is facilitated by the glow reaction (rather than a brief flash) along with the higher enzymatic activity. Three different split formats have been developed: a hybrid, a two-component, and a three-component assay. As shown in Table 3, there are several advantages and disadvantages of LIPS versus the mix and read technologies. The transitional hybrid assay using the HiBiT-antigen fusion and LgBiT fragment along with protein G beads does not offer any significant advantage over standard LIPS since it still requires a washing step of the beads [74]. The standard LIPS assay, and to some extent the hybrid, can be developed rapidly for any target antigen with minimal effort. In contrast, the two- and three-component assays require significant development time for each antigen. This is due to the time necessary for the molecular design of optimum configuration and orientation of the antigen targets with the Nano fragments, assay component titration for optimization, and potential background issues (Table 3). The three-component assay also requires multiple serum dilutions to achieve a final measurement value [78]. Based on these limitations, the mix and read assays will be primarily developed against known immunogenic target proteins and not used for the discovery of new antigens. Nonetheless, the key advantages of these assays are the limited sample processing and equipment required, only a luminometer is needed for reading. One barrier to the rapid wide-spread development of the mix and read assays based on split Nano is the need for highly purified protein preparations produced in bacteria or mammalian cells (Table 3). Purification, quantitation, and standardization of the component are needed. Unlike conventional LIPS, the amount and purity of each protein component need to be determined by conventional techniques (e.g., Western blot), whereas the input probe concentration in LIPS can be assessed with a simple luminometer reading of a crude cellular extract. Nonetheless, once all the elements are produced, they can be employed in point-of-care settings. A last limitation of the mix and read formats involves the likely difficulty in generating assays to large antigenic targets (Table 3). For example, a LIPS PLA2R autoantibody detection assay works well without purification of the luciferase-labeled antigen to detect autoantibodies against this large protein of approximately 1400 amino acids [29]. Conversely, it would be difficult to produce and purify from *E. coli* such a large protein and it would unlikely be folded properly. Nevertheless, these considerations should not impede the development of mix and read assays for other appropriate smaller targets, many of which have substantial clinical importance.

It is likely that the mix and read technologies will continue to evolve in the clinical arena. One major commercial opportunity would be to develop a large panel of antigenic targets in the mix and read format that is already known to have high diagnostic performance in the standard LIPS assay. These include a variety of infectious agents such as HIV, HCV, and HBV, as well as autoantigens highly useful for the diagnosis and monitoring of various autoimmune diseases. In summary, mix and read assays based on the reconstitution of two or three Nano fragments have proven to be a viable immunoassay format for antibody detection. The ability to couple this format to detection by a hand-held luminometer may facilitate POC testing. Based on the compelling potential of these mix and read assays, a large panel of these immunoassays could be used in parallel to provide fast antibody-based information for diagnoses, treatment monitoring, and comprehensive health monitoring. Lastly, one potential future commercial development would be to develop an instrument that could simultaneously process and read multiple individual mix and read tests for different autoimmune and infectious diseases at the same time, analogous to a device used for array-based comprehensive molecular pathogen detection [83].

## Figures and Tables

**Table 1:** Luciferases used for Antigen Fusion for LIPS testing.

Luciferase	*Renilla* Luciferase (Ruc)	*Gaussia* Luciferase (GLuc)	Nanoluciferase (Nano)
**Size**	36 kDa	20 kDa	19 kDa
**Substrate**	Coelenterazine	Coelenterazine	Furimazine
**Signal Type**	Flash	Flash	Glow
**Location of Antigen Target Fusion**	C-terminal	N- and C-terminal	N- and C-terminal
**Adapted to Hand-held Luminometer**	No	No	Yes

**Table 2:** Serology-Enabled Discovery of New Autoimmune Diseases.

New Autoimmune Disease	Significance
Opportunistic Infection (OI) in patients with Thymoma Cancer	Detection of autoantibodies against IFN-α1, IL-12 and other cytokines associated with OI pts [40]
Autoimmune associated disseminated non-tuberculosis mycobacterial infection (dNTM)	Autoantibodies against IFN-γ associated with mycobacterial infection [41]
Autoimmune hyperphosphatemia	First case of hyperphosphatemia due to autoantibodies against FGF23 [45]
Autoimmune hypoparathyroidism	Blocking autoantibodies against PTH1R [46]

**Table 3:** Advantages and Disadvantages of LIPS and Split Luciferase Technologies.

Features	LIPS	Split Fab	SATiN
**Luciferase used**	Ruc, Gluc, and Nano	Split Nano	Split Nano
**Number of protein components**	One	Two	Three
**Assay development**	Simple	Complex	Complex
**Recombinant Expression**	Crude cell extract	Purified	Purified
**Protein size constraints**	No	Yes	Yes
**Hands on time per 96 samples**	~30 min	~15 min	~30 min
**Washing steps**	Yes	No	No
**Antigen discovery**	Yes	No	No

## Data Availability

Not applicable.
